# Risk factors for *Entamoeba histolytica *infection in an agricultural community in Hanam province, Vietnam

**DOI:** 10.1186/1756-3305-4-102

**Published:** 2011-06-10

**Authors:** Phuc Pham Duc, Hung Nguyen-Viet, Jan Hattendorf, Jakob Zinsstag, Phung Dac Cam, Peter Odermatt

**Affiliations:** 1 Department of Epidemiology and Public Health, Swiss Tropical and Public Health Institute, P.O. Box CH-4002, Basel, Switzerland; 2 University of Basel, P.O. Box CH-4003, Basel, Switzerland; 3 National Institute of Hygiene and Epidemiology (NIHE), 1 Yersin, Hanoi, Vietnam; 4 Department of Environmental Health, Hanoi School of Public Health (HSPH), 138 Giang Vo, Hanoi, Vietnam; 5 Swiss Federal Institute of Aquatic Science and Technology (EAWAG), Sandec - Department of Water and Sanitation in Developing Countries, Dübendorf, Switzerland

## Abstract

**Background:**

*Entamoeba histolytica *is an important protozoan intestinal infection in resource-poor settings, including Vietnam. The study objective was to assess risk factors of *E. histolytica *infection in a community in Vietnam, where wastewater and human excreta are used in agriculture. A case-control study was conducted among residents of Hanam province, Northern Vietnam. Cases (n = 46) infected with *E. histolytica *and non-infected controls (n = 138) were identified in a cross-sectional survey among 794 randomly selected individuals and matched for age, sex and place of residence. Potential risk factors including exposure to human and animal excreta and household wastewater were assessed with a questionnaire.

**Results:**

People from households with an average socio-economic status had a much higher risk of *E. histolytica *infection (odds ratio [OR]=4.3, 95% confidence interval [CI]: 1.3-14.0) compared with those from households with a good socioeconomic status. Those individuals who never or rarely used soap for hand washing had a 3.4 times higher risk for infection (OR=3.4, 95% CI: 1.1-10.0), compared to those who used always soap. In contrast, none of the factors related to use of human or animal excreta was statistically significant associated with *E. histolytica *infection. People having close contact with domestic animals presented a greater risk of *E. histolytica *infection (OR = 5.9, 95% CI: 1.8-19.0) than those without animal contact. *E. histolytica *infection was not associated with direct contact with Nhue river water, pond water and household's sanitary conditions, type of latrine or water source used.

**Conclusions:**

Our study suggests that in settings where human and animal excreta and Nhue River water are intensively used in agriculture, socio-economic and personal hygiene factors determine infection with *E. histolytica*, rather than exposure to human and animal excreta in agricultural activities.

## Background

Amoebiasis caused by the intestinal parasite* Entamoeba histolytica*, has an estimated worldwide prevalence of 500 million infected people and is responsible for 40,000 - 100,000 deaths each year. It is an important health problems, especially in developing countries [1,2]. The incidence rate of *E. histolytica*-associated diarrhoea was 0.08/child-year [3]. The rate of infection by *E. histolytica *differs among countries, socio-economic and sanitary conditions and populations [4]. It is highly endemic throughout poor and socio-economically deprived communities in the tropics and subtropics. Environmental, socio-economic, demographic and hygiene-related behaviour is known to influence the transmission and distribution of intestinal parasitic infections [5]. A study in Brazil identified place of residence, age, ingestion of raw vegetables and drinking water quality as important risk factors [6].

Wastewater and human and animal excreta are used as fertilizer for a wide variety of crops, and 10% to 30% increases in crop yields have been reported [7]. The use of wastewater and human and animal excreta in agriculture and aquaculture continues to be common in China, South and South East Asia as well as various areas in Africa [8-10] in particular where water scarcity is becoming more severe. The main sources of water for irrigation in Vietnam are fresh water, wastewater and ground water. In Hanoi about 80 percent of vegetable production is from urban and peri-urban areas irrigated with diluted wastewater [11]. The use of household sewage, and human and animal excreta in agriculture and aquaculture has a long tradition in Vietnam [12]. Despite the potential health risk for intestinal disease of using excreta and animal waste in agriculture [13], 85% of farmers in northern provinces of Vietnam regularly use human excreta in agriculture [14]. Another study in Vietnam on helminth infections among people exposed to wastewater and human excreta has showed that wastewater exposure was not an important risk factor for parasite infection but that the lack of sanitation facilities and the use of fresh or inadequately composted excreta as fertilizers in agriculture increased the risk of parasite infection [15]. A study in Hanoi, Vietnam, on the epidemiology and aetiology of diarrhoeal diseases in adults engaged in wastewater-fed agriculture and aquaculture has showed that the diarrhoeagenic *Escherichia coli *and *E. histolytica *were the most common pathogens [16,17].

To further understand the transmission of *E. histolytica *infection, we conducted a case-control study to assess the importance of handling practices of human and animal excreta and wastewater use in irrigation in agriculture and aquaculture, in relation to other potential risk factors, including sanitary conditions, drinking water, food consumption, and personal hygiene practices.

## Methods

### Study sites

The study was carried out in Nhat Tan and Hoang Tay communes in Kim Bang district, Hanam province (20.32° N, 105.54°E), Northern Vietnam located about 60 km south of Hanoi. The number of inhabitants is about 10,500 (2,600 households) and 5,700 (1,500 households) in Nhat Tan and Hoang Tay communes, respectively. Most households have livestock in their compounds. The residential areas are in the vicinity of fields used for agriculture (rice and vegetables) and aquaculture (fish breeding). The rice fields and local ponds cover about 50% of the surface. The two communes border the Nhue River. Hanoi City's wastewater from households, industry and other sources such as hospitals is directly and untreated discharged into the river [18]. The Nhue River water is used for crop irrigation and to feed fishponds. Several pump stations located along the river and a system of open and closed canals distribute the water to the local fields and fish ponds. Wastewater from households (grey water from kitchens and bathrooms, and effluent from septic tanks and sanitation facilities) is discharged into the small irrigation canals. The area has two main rice production cycles per year, one called "spring season" from January to June and the other "summer season" from July to October. People also grow vegetables which are eaten raw or cooked by the local population or sold to neighbouring towns and Hanoi. Human excreta are used as fertiliser in Hanam as in many other places in Northern and Central Vietnam. In general, excreta from double or single vault latrines are not or only partially composted. The composting procedure does not fully respect the composting guidelines set out by the Vietnamese Ministry of Health which imposes a minimum of 6 months [19]. In practice, farmers utilise the latrine night soil to fertilise crops whenever they need it in the fields, which results often in a shorter storage period than the regulatory 6 months; personal protective measures to prevent contamination are often lacking.

### Study design

This study carried out in August 2008 followed the logic of a community based case-control study. A subject was defined as case if diagnosed with an *E. histolytica *infection (at least one of two stool samples positive for *E. histolytica*). Controls were subjects negative for *E. histolytica *in two stool examinations and matched for sex, age groups (i) under 6 years, (ii) 6-15 years, (iii) 16-30 years, (iv) 31-45 years, (v) 45-60 years, and (vi) over 60 years, and place of residence (same commune but different household).

### Ascertainment of cases and controls

The cases and controls were identified in a large cross-sectional household survey on intestinal parasitic infections. All patients infected with *E. histolytica *were enrolled as cases. Controls were selected randomly among the non-infected individuals.

Fifteen villages in Nhat Tan and 10 villages in Hoang Tay communes were selected to participate in the cross-sectional study. Households were randomly selected from the household list provided by the Communal People's Committee. Out of the 4282 households living in the two communities, 270 households were selected using random numbers. All household members above 12 months of age were enrolled.

Two stool samples were collected from each enrolled individual on two consecutive days. Each family member was provided with a labelled plastic container to collect a stool sample on the following day (day 1) by trained personnel. When first container with stool was collected, a second labelled container was provided for the stool of the following day (day 2). Samples were transported to the laboratory of the Parasitological Department in Hanoi Medical University within 4 hours after collection and stored at 4-8^°^C until analysis.

### Laboratory procedures

The formalin-ether concentration technique (FECT) was used for detecting *E. histolytica *[20]. In brief, the preparation process was as follows: a stool sample of approximately 1 gram was places into a tube containing 10 mL of formalin. The sample was mixed thoroughly and vigorously, and then the stool solution was filtered using a funnel with gauze and centrifuged for 1 minute at 447 × g. Supernatants were removed with a pipette, and 7 mL saline solution were added and mixed with a wooden stick. 3 mL Ether were then added and the tubes closed with rubber stoppers and shaken well (about 30 seconds). The rubber stoppers were then carefully removed and the tubes were centrifuged for 5 minutes at 447 × g. The supernatant was discarded and the entire sediment was examined for the presence of protozoa using a microscope at a magnification of 500x.

### Sample size

The sample size was calculated for the matched case-control study with a ratio between case and control groups of 1:3 [21]. To detect - at a 95% confidence level - an odds ratio (OR) = 2.5 with a power of 80% and an expected frequency of exposure to excreta and wastewater in the control group of 30%, we require sample sizes of 52 cases and 156 controls.

### Data collection

A questionnaire with six sections was administered to all cases and controls: (i) general demographic information and socio-economic status (SES): age, gender, educational level, occupation, household's economic status was assessed with a list of indicators which included surface of household's rice field and fish ponds, number of animals (pigs, buffalos, chickens, ducks, cows, dogs and cats), housing characteristics (building materials, number of bedrooms), and household assets (motorbike, bicycle, refrigerator, television, radio, telephone, bed, cupboard, electric fan and electronic devices); (ii) household sanitary conditions: general sanitary conditions was assessed by following indicators: the condition and location of the household's latrine (smell, flies, broken door, mud around the latrine); water storage container with cover and wastes (domestic waste, human/animal faeces) in the yard, type of latrine, type of water used in household and direct contact with animals in the household (i.e. pig, chicken, duck, dog and cat); (iii) exposure to human and animal excreta at home and in the fields; (iv) exposure to water from the Nhue River and local ponds, and irrigation water; (v) personal hygiene habits and practices: use personal protection during field work (gloves, boots, etc.), bathing and hand washing after work with or without soap, eating habits, eating leftovers from day before, and source of drinking water; (vi) information related to gastrointestinal symptoms: vomiting, nausea, abdominal pain, watery stools, blood/mucus stools and loose stools.

The questionnaire was developed in English, translated to Vietnamese and pre-tested in villages close to Hanoi. After adaptation the questionnaire was used in a face-to-face interview conducted by trained and experienced research assistants. The main researcher accompanied each assistant to three households and supervised him/her to make sure that the procedure was being precisely followed. Each interview took approximately 45 minutes.

### Data management and analysis

Data was double-entered in a Microsoft Access database and validated. Analysis was performed using STATA version 10.1 (StataCorp, College Station, TX, USA).

Statistical analysis for the matched case-control study was conducted as follows. First, an univariable conditional logistic regression analysis was carried out to associate potential risk factors with infection status (outcome) for which matched OR and its 95% confidence interval (CI) and P-value were calculated. Then, variables with P < 0.2 in the univariable analysis were included in the multivariable conditional logistic regression analysis [22]. Variables related to personal hygiene behaviour were highly inter-correlated. Therefore, we included only one variable (hand washing with soap) in the multivariable model to avoid collinearity.

SES and sanitary conditions in the household were calculated according to an asset-based method [23,24]. In brief, indicator data were defined by principal component analysis (PCA), with missing values being replaced with the mean value of the respective asset; all assets had a dichotomous character. SES and sanitary conditions in the household were categorized into three levels as good, average, and poor according to their cumulative standardized asset scores.

### Ethical considerations

Before field work the authorities in the Provincial Health Office and the District Health Office were informed and asked for permission. Detailed information on study objectives and procedures was provided and working authorisation obtained. Written informed consent was obtained from each individual prior to enrolment. The Ethical Research Committee at the National Institute of Hygiene and Epidemiology (NIHE, number 149/QĐ-VSDTTU-QLKH, 22 April 2009), Vietnamese Ministry of Health and the Ethic Commission of the State of Basel (EKBB, number 139/09, 11 May 2009) approved the study.

## Results

### Description of cases and controls

We identified and enrolled 46 cases and 138 controls. The mean age for cases and controls was 34 years (SD 2.8 years, range: 3-83 years) and 36 years (SD 1.3 years, range: 5-87 years), respectively. Thirty-one cases (67.4%) were found in Nhat Tan and 15 cases (32.6%) in Hoang Tay commune. The mean family size for cases and controls was 4.1 (SD 1.6) and 4.2 (SD 1.3) persons, respectively, and was not statistically significantly different (P = 0.46).

Only few study participants reported gastrointestinal symptoms: eleven cases (23.9%) and 17 controls (12.3%). There was no significant statistical difference between the two groups (P > 0.20). The gastrointestinal symptoms were fever (2 cases, 1 control), nausea (1 case, 0 control), abdominal pain (4 cases, 9 controls), and watery stools (4 cases, 7 controls).

### Risk factors for*E. histolytica *infection

The results of the univariable and multivariable conditional logistic regression analysis are presented in Table 1 and Table 2, respectively.

**Table 1:** Risk factors for *E. histolytica* infection in Hanam province, Vietnam (univariable conditional logistic regression analysis).

Variables	Case	Control			
	N (%)	N (%)	Matched OR	95% CI	P-value
**1. Socio-economic status**					
Educational level					
High school	6 (13.0)	17 (12.0)	Reference		
Secondary school	24 (52.2)	79 (57.3)	0.8	0.7-2.7	0.76
Primary school	16 (34.8)	42 (29.8)	1.1	0.3-3.8	0.87
Occupation					
Non agricultural work	16 (34.8)	45 (32.6)	Reference		
Agricultural work	30 (65.2)	93 (67.4)	0.8	0.3-2.2	0.70
Household's economic status overall					
Good	8 (17.4)	53 (38.4)	Reference		
Average	22 (47.8)	39 (28.3)	3.8	1.5-9.8	0.01
Poor	16 (34.8)	46 (33.3)	2.4	0.9-6.4	0.08
**2. Household sanitary and hygiene conditions**					
Household's sanitary conditions overall					
Good	20 (43.5)	40 (29.0)	Reference		
Average	12 (26.1)	50 (36.2)	0.5	0.2-1.1	0.08
Poor	14 (30.4)	48 (34.8)	0.6	0.3-1.3	0.21
Type of latrine in the household					
Water latrine (septic tank, biogas)	15 (32.6)	47 (34.1)	Reference		
Dry latrine (single or double vault)	29 (63.0)	87 (63.0)	1.1	0.5-2.2	0.89
No latrine	2 (4.4)	4 (2.9)	1.7	0.2-11.2	0.61
Household use of tap water					
No	24 (52.2)	89 (64.5)	Reference		
Yes	22 (47.8)	49 (35.5)	1.7	0.9-3.4	0.13
Household use of tube well water					
No	16 (34.8)	53 (38.4)	Reference		
Yes	30 (65.2)	85 (61.6)	1.2	0.6-2.5	0.64
Household use of rainwater					
No	5 (10.9)	12 (8.7)	Reference		
Yes	41 (89.1)	126 (91.3)	0.8	0.3-2.4	0.66
Close contact with domestic animals in household					
No	9 (19.6)	42 (30.4)	Reference		
Yes	37 (80.4)	96 (69.6)	1.9	0.8-4.4	0.15
**3. Exposed to human and animal excreta**					
Composting of human excreta in the household					
No	22 (47.8)	60 (43.5)	Reference		
Yes	24 (52.2)	78 (56.5)	0.8	0.4-1.7	0.59
Use of human excreta for application in field					
No	18 (39.1)	62 (44.9)	Reference		
Yes	28 (60.9)	76 (55.1)	1.3	0.6-2.6	0.48
Handling human excreta in field work					
No	22 (47.8)	61 (44.2)	Reference		
Yes	24 (52.2)	77 (55.8)	0.8	0.4-1.8	0.61
Compound with animal husbandry					
No	6 (13.0)	25 (18.1)	Reference		
Yes	40 (87.0)	113 (81.9)	1.5	0.6-4.0	0.42
Composting of animal excreta in the compound					
No	31 (67.4)	79 (57.3)	Reference		
Yes	15 (32.6)	59 (42.7)	0.7	0.3-1.3	0.23
Use of animal excreta as fertiliser in the fields					
No	25 (54.3)	69 (50.0)	Reference		
Yes	21 (45.7)	69 (50.0)	0.8	0.4-1.7	0.59
Handling animal excreta in field work					
No	31 (67.4)	69 (50.0)	Reference		
Yes	15 (32.6)	69 (50.0)	0.5	0.2-0.9	0.03
**4. Exposed to water from Nhue river and local pond**					
Direct contact with Nhue river water during field work					
No	30 (65.2)	72 (52.2)	Reference		
Yes	16 (34.8)	66 (47.8)	0.6	0.3-1.2	0.12
Use local pond for fishing, bathing, washing					
No	32 (69.6)	95 (68.9)	Reference		
Yes	14 (30.4)	43 (31.1)	1.0	0.5-2.1	0.92
Use Nhue river water to irrigate fields					
No	1 (2.2)	13 (9.4)	Reference		
Yes	45 (97.8)	125 (90.6)	4.6	0.6-35.4	0.15
**5. Personal hygiene habits**					
Use protective measures (gloves, boots and face mask) at work					
No	28 (60.9)	63 (45.6)	Reference		
Yes	18 (39.1)	75 (54.4)	0.5	0.3-1.1	0.07
Showering, bathing (with soap) after field work					
Frequently	9 (19.6)	48 (34.8)	Reference		
Sometimes	19 (41.3)	49 (35.5)	2.2	0.9-5.5	0.09
Rarely	18 (39.1)	41 (29.7)	2.3	1.0-5.6	0.06
Washing hands after field work					
Frequently	30 (65.2)	108 (78.3)	Reference		
Sometimes	3 (6.5)	9 (6.5)	1.6	0.4-7.0	0.50
Rarely	13 (28.3)	21 (15.2)	3.4	1.2-10.0	0.02
Washing hands with soap after field work					
Frequently	9 (19.6)	50 (36.2)	Reference		
Sometimes	14 (30.4)	43 (31.2)	1.8	0.7-5.1	0.24
Rarely	23 (50.0)	45 (32.6)	3.0	1.2-7.4	0.02
Eating leftover food from day before					
No	13 (28.3)	42 (30.4)	Reference		
Yes	33 (71.7)	96 (69.6)	1.1	0.5-2.3	0.78
Eating raw vegetables the day before					
No	44 (95.6)	130 (94.2)	Reference		
Yes	2 (4.4)	8 (5.8)	0.72	0.1-3.7	0.70
Water source for drinking					
Rainwater	42 (91.3)	129 (93.5)	Reference		
Tube well water	4 (8.7)	9 (6.5)	1.4	0.4-4.9	0.61

**Table 2:** Risk factors for *E. histolytica* infection in Hanam province, Vietnam (multivariable conditional logistic regression analysis).

Risk factors	Matched OR	95% CI	P-value
Household's socioeconomic status (*versus *good)			
- average	4.3	1.3-14.0	0.02
- poor	2.2	0.6-7.4	0.22
			
Household's sanitary conditions (*versus *good)			
- average	0.8	0.3-2.3	0.68
- poor	1.6	0.6-4.6	0.38
Household with tap water (yes *versus *no)	1.3	0.5-3.1	0.57
Close contact with domestic animals in household (yes *versus *no)	5.9	1.9-18.9	0.003
			
Handling animal excreta in field work (yes *versus *no)	0.2	0.1-0.7	0.01
			
Direct contact with Nhue river water during field work (yes *versus *no)	0.4	0.1-1.1	0.07
Use of Nhue river water to irrigate fields (yes *versus *no)	3.7	0.4-33.1	0.24
			
Washing hands with soap after field work (*versus *frequently)			
- sometimes	1.7	0.5-5.8	0.40
- rarely	3.4	1.1-10.0	0.03

Among the indicators describing the general and socio-economic status of the family the general socio-economic status was strongly associated with the *E. histolytica *infection. Participants who lived in households with an average and poor SES had a 3.8 (95% CI: 1.5-9.8) and 2.4 (95% CI: 0.9-6.4) higher risk of infection with *E. histolytica *than those living in households with a good status. The multivariable conditional logistic regression analysis confirmed this finding with the same trend (OR = 4.3, 95% CI: 1.3-14.0). Although in uni- and multivariable analysis the risk increase was high with decreasing general SES, statistically significant risk increase was found only for average versus good SES (i.e. OR = 4.3, P = 0.02, Table 2).

Cases and control did not differ in educational levels. Furthermore, no statistically significant difference was found in occupation. Approximately two third of both groups were farmers (65.2% of cases *versus *67.4% of controls, P = 0.79). Sixteen cases (34.8%) and 45 controls (32.6%) were officers in public services such as teachers, health workers, or small traders, or were retired or working at home.

The sanitary conditions of the household were described with an overall assessment indicator, the type of latrines present and type of water used in the household. In none of these analyses was a significant association between the indicators and the infection status with *E. histolytica *found (Table 1 and 2). However, close contact with domestic animals in the household resulted in a statistical significant two-fold and six-fold risk increase for a *E. histolytica *infection in the univariable (OR = 1.9, 95% CI: 0.8-4.4) and multivariable analysis (OR = 5.9, 95% CI: 1.9-18.9).

In univariable analysis, none of the variables related to human excreta showed an increased risk of *E. histolytica *infection. For example, composting of human excreta (OR = 0.8, 95% CI: 0.4-1.7), or use of human excreta as fertiliser for application in field (OR = 1.3, 95% CI: 0.6-2.6), or handling human excreta in field work (OR = 0.8, 95% CI: 0.4-1.8). No association was found for composting of animal excreta (OR = 0.7, 95% CI: 0.3-1.3) and use of animal excreta as fertiliser for application in field (OR = 0.8, 95% CI: 0.4-1.7). On the contrary, handling animal excreta in the field was found to be a protective factor in the univariable (OR = 0.5, 95% CI: 0.2-0.9) and multivariable analysis (OR = 0.2, 95% CI: 0.1-0.7).

Direct contact with Nhue River water during field work resulted in a substantial risk reduction in the uni- (OR = 0.6, 95% CI: 0.3-1.2) and multivariable analysis (OR = 0.4, 95% CI: 0.1-1.1). Using the Nhue River water to irrigate fields increased the risk (OR = 4.6 and OR = 3.7 in the uni- and multivariable analysis, respectively) but it was not statistically significant. There was no risk change for *E. histolytica *infected individuals associated with close contact and use of local ponds (OR = 1.0, 95% CI: 0.5-2.1, P = 0.92).

Risk changes were observed for variables related to personal hygiene. Using personal protective conditions during field work such as gloves and boots reduced the risk (OR = 0.5, 95% CI: 0.3-1.1) and omitting to bath and shower after field work increased the risk (OR = 2.3, 95% CI: 1.0-5.6) for an infection with *E. histolytica*. However, these associations were not statistically significant. Omitting to wash the hands was a significant risk. E.g., People who rarely washed their hands with soap after field work had a large risk increase of an *E. histolytica *infection (OR = 3.0, 95% CI: 1.2-7.4) compared to those who frequently wash their hand with soap after work. This risk increase remained statistically significant in the multivariable analysis (OR = 3.4, 95% CI: 1.1-10.0).

Consuming leftover foods from the day before (OR = 1.1, 95% CI: 0.5-2.3), eating raw vegetables (OR = 0.7, 95% CI: 0.1-3.7) and type of water source used for drinking water (OR = 1.4, 95% CI: 0.4-4.9) were not associated with *E. histolytica *infection.

## Discussion

We have studied risk factors associated with *E. histolytica *infection in a semi-rural community where human and animal excreta are intensively used as fertiliser in agriculture and where household wastewater is directed into irrigation channels. We identified lower economic status of households (OR = 4.3), poor hand washing practices after work (OR = 3.4) and close contact with animals in the household (OR = 5.9) as major risk factors for *E. histolytica *infection. None of the factors measuring exposure to human and animal excreta such as composting excreta in the household or using excreta as fertilisers in the field resulted in an increased risk. On the contrary, those who reported handling animal excreta during field work had a substantial risk reduction (OR = 0.2). In addition, close and frequent exposure to Nhue River water reduced the risk (OR = 0.4).

*E. histolytica *developing in humans is transmitted directly following faecal-oral transmission routes. The risk pattern identified in our study follows this logic. In particular, the transmission routes via contaminated hands play a major role, documented in our study with a more than three- fold risk increase if hands are not washed properly. In contrast, the transmission routes via contaminated food are not of relevance. We did not find any association between an *E. histolytica *infection and consumption of raw vegetables, leftover food from previous days and different types of drinking water. Similar observations were made by Nyarango and colleagues in Kenya [25]. In addition, we observed in our study area that vegetables were grown usually in a garden, very close to the house where wastewater and human excreta were not likely to be used often for irrigation and as fertilizers, probably due to the smell of human excreta. Furthermore, it was frequently seen that vegetables are properly washed before they are consumed. Indeed, a study in Iran indicated that no parasitic contamination was found on any of the washed samples of vegetables [26,27].

Interestingly, close contact with domestic animals was associated with an important risk increase. This finding is somehow difficult to explain. But it is well possible that cysts of *Entamoeba *deposited on the surface (fur) of the animals during close contact with humans and then later transmitted to a next person. In order to support this hypothesis, the presence of *Entamoeba *cysts in fur must be documented. Unfortunately, we could not conduct this verification during our field work.

Our study showed that agricultural field practices which involve handling of excreta of humans and animals are not relevant for the transmission although a considerable *Entamoeba *infection prevalence was documented in the faeces. Although *E. histolytica *cysts are quite resistant, they perish in human excreta within a short time period of storage or composting. Protozoan cysts, including those of *G. intestinalis *and *E. histolytica*, are unlikely to survive more than 10 days in soil as they are susceptible to desiccation [28]. On the contrary, we found that those handling animal excreta in the field had a significantly lower risk for an *E. histolytica *infection than those who have no contact with animal excreta. Several points are important with regard to this result. First, animals do not harbour *E. histolytica *infections, it is rarely found in domestic animals, including dog and cat [29,30] and therefore, it is unlikely that cysts are present in the stool. Secondly, all excreta is stored before being utilised in agriculture. The time period and conditions of the storage often do not meet full safety regulations [31]. However, they are sufficient to eliminate an important portion of the infectious agents, including *E. histolytica *cysts [13,28]. Thirdly, those handling animal excreta are more likely to use personal protective measure and wash their hands with soap after work, i.e. in our study area; the Nhue River is an excellent opportunity for that as it is situated next to the agricultural land. Indeed, 96.4% of those handling animal excreta washed their hands after work compared to 61.0% of those who did not handle animal excreta.

The agricultural area of our study borders the Nhue River. Water from the Nhue River is used intensively for irrigation of fields and personal hygiene of farmers during field work. We found that intensive contact with Nhue river water during field work reduced the risk. This finding is to some degree in contradiction to the results of the study in Hanoi where diarrhoea episodes were significantly associated with contact with river water [16,17]. However, it must be noted that our study area is at a considerable distance to Hanoi and important agglomerations (60 km) where substantial contamination takes place. Hence, the concentration of infectious agents are diluted to a much higher degree [32].

Our study has some limitations. First, we had a relative small number of cases which resulted in a relative small overall sample size. Changes of exposure in a few cases may result in a risk change which is statistically not significant. For example, we found a statistical significant increased risk for households with average compared with good socio-economic status. However, those participants with a poor SES had an increased risk which was not statistically significant. Also, the risk increase observed for those who use Nhue River water to irrigate field was not statistically significant. The small sample size could be a reason for this statistically non-significant observation. Other studies could show an increased risk of protozoan infection associated with Nhue River water [33]. Secondly, in our dataset the variables describing practices and habits of personal hygiene were highly correlated. Therefore, we could retain only one variable for the multivariable analysis. As a consequence, we could not perform a fine tuned multivariable analysis in which the effects of the different hygiene practices could be directly compared.

The association between infection and households' SES indicated that the participants living in households with an average SES presented a more than four fold risk increase (OR = 4.3, 95% CI: 1.3-14.0) compared to those living in households with a good SES. This finding is similar to that found in previous epidemiological studies indicating that unsanitary conditions and low SES were significant risk factors for *E. histolytica *infection [34-37].

However, in our study, there was no significant link between* E. histolytica *infection and participants' level of education. Our study population was relatively well educated. Two-third of our study participants finished secondary or high school and were generally very knowledgeable. A similar observation was made in Pakistan [38].

The fact that the households' water source was not a risk factor for *E. histolytica *infection is not surprising. Indeed, it was commonly observed that boiled rainwater was used for drinking in almost all study households. Nevertheless, a study from central Vietnam showed that river water may be an important source of *E. histolytica *infection [39]. An other study in Thailand found that the lack of regular water-treatment practices was also a risk factor [40].

The diagnostic method we used (FECT) does not allow the distinction between pathogenic *E. histolytica *from non-pathogenic *E. dispar *[41] which can be made by isoenzyme analysis and molecular technique [42]. Therefore, whenever *E. histolytica *is named in this article, it can not be excluded that it is *E. dispar*. *E. histolytica *infection, and resulting intestinal disease and liver abscesses are a public health concern in many tropical areas, including Vietnam. In our study we diagnosed among 794 randomly selected individuals 46 (5.8%) infected persons. Virtually all of them were asymptomatic but contribute to transmission. Even higher prevalence rates were observed in different parts of Vietnam, e.g. in a suburb of Hanoi and in Hue where 10.0% and 11.2% were infected [16,39].

## Conclusions

Our study documents that agricultural practice in which human and animal excreta and household waste water are used as fertiliser and for irrigation are not relevant for the transmission of *E. histolytica*. It confirms that in these settings other transmission routes such as contaminated hand are of importance and provides further arguments that basic personal hygiene measures such as hand washing with soap must be further promoted.

## Conflict of interest

The authors declare that they have no competing interests.

## Authors' contributions

PPD, HNV, JZ and PO planned the study and designed the protocols. PPD, HNV and PDC conducted the field study and supervised the study programme, including the collections of stool samples and data from the questionnaire interviews, as well as the management of collected data. PPD and HNV supervised all the laboratory work. PPD, JH, PO and HNV carried out the data analysis and interpretation. PPD, HNV and PO prepared the first draft of the manuscript and all authors revised the manuscript critically. All authors read and approved the final version of the manuscript.

## References

[B1] WHO WHO/PAHO/UNESCO report. A consultation with experts on amoebiasis. Mexico City, Mexico 28-29 January, 1997 Epidemiol Bull 1997 18 13 14 9197085

[B2] Sebastiaan JvHJV Stark DJ Fotedar R Marriott D John TEJT Harkness JL Amoebiasis: current status in Australia M J A 2007 186 412 416 10.5694/j.1326-5377.2007.tb00975.x 17437396

[B3] Haque R Mondal D Kirkpatrick BD Akther S Farr BM Sack RB Petri WA Epidemiologic and clinical characteristics of acute diarrhea with emphasis on Entamoeba histolytica infections in preschool children in an urban slum of Dhaka, Bangladesh Am J Trop Med Hyg 2003 69 398 405 14640500

[B4] Al-Harthi S Jamjoom M Diagnosis and Differentiation of Entamoeba infection in Makhah Al Mukarramah using microscopy and stool Antigen Detection Kits W J Med Sci 2007 2 15 20

[B5] Norhayati M Fatmah MS Yusof S Edariah AB Intestinal parasitic infections in man: a review Med J Malaysia 2003 58 296 305 14569755

[B6] Benetton ML Goncalves AV Meneghini ME Silva EF Carneiro M Risk factors for infection by the Entamoeba histolytica/E. dispar complex: an epidemiological study conducted in outpatient clinics in the city of Manaus, Amazon Region, Brazil Trans R Soc Trop Med Hyg 2005 99 532 540 10.1016/j.trstmh.2004.11.015 15869773

[B7] Asano T Wastewater reclamation and reuse 1998 Technomic Publishing Company Inc

[B8] Drechsel P Scott CA Raschid-Sally L Redwoodand AB Wastewater irrigation and health - Assessing and mitigating risk in low-income countries 2010 Sterling, VA, London: Earthscan

[B9] Cross P Health Aspects of Nightsoil and Sludge Use in Agriculture and Aquaculture-Part I: Existing Practices and Beliefs in the Utilisation of Human Excreta International Reference Center for Waste Disposal (No 04/85), Dubendorf, Switzerland 1985

[B10] Timmer L Visker C Possibilities and Impossibilities of the use of human excreta as fertiliser in agriculture in sub-Saharan Africa Royal Tropical Insitute and University of Amsterdam, The Netherlands 1998

[B11] Lai T Perspectives of peri-urban vegetable production in Hanoi 2002

[B12] Shuval HI Adin A Fattal B Rawitz E Yekutiel P Wastewater irrigation in developing countries: Health effects and technical solutions World Bank Technical Paper Number 51 The International Bank for Reconstruction and Development/The World Bank 1986

[B13] WHO Guidelines for the safe use of wastewater, excreta and greywater - Volume 4: Excreta and Greywater use in agriculture 2006 Geneva, Switzerland: World Health Organization

[B14] Phuc PD Konradsen F Phuong PT Cam PD Dalsgaard A Practice of using human excreta as fertilizer and implications for health in Nghean Province, Vietnam Southeast Asian J Trop Med Public Health 2006 37 222 229 16771238

[B15] Do TT Molbak K Phung DC Dalsgaard A Helminth infections among people using wastewater and human excreta in peri-urban agriculture and aquaculture in Hanoi, Vietnam Trop Med Int Health 2007 12 Suppl 2 82 90 18005319 10.1111/j.1365-3156.2007.01945.x

[B16] Do TT Bui TT Molbak K Phung DC Dalsgaard A Epidemiology and aetiology of diarrhoeal diseases in adults engaged in wastewater-fed agriculture and aquaculture in Hanoi, Vietnam Trop Med Int Health 2007 12 Suppl 2 23 33 18005312 10.1111/j.1365-3156.2007.01938.x

[B17] Hien BTT Trang DT Schuetz F Cam PD Molbak K Dalsgaard A Diarrhoeagenic Escherichia coli and other causes of childhood diarrhoea: a case-control study in children living in a wastewater-use area in Hanoi, Vietnam Journal of Medical Microbiology 2007 56 1086 1096 10.1099/jmm.0.47093-0 17644717

[B18] Ministry of Natural Resources and Environment Improving Water Quality in the Day/Nhue River Basin: Capacity Building and Pollution Sources Inventory ADB/MARD/MONRE Project 3892-VIE 2007

[B19] Ministry of health Regarding issuing of the sector standards. Hygiene standards of various type of latrines. Decision of the Ministry of Health N0. 08/2005/QD.BYT 2005

[B20] Lynne SG Clinical Microbiology Procedures Handbook 2007 Washington, D.C.: ASM Press

[B21] Schlesselman JJ Case control studies, in Design, Conduct, Analysis 1982 Oxford University Press

[B22] Hosmer DW Jr Lemeshow S Applied Logistic Regression 1989 New York: John Wiley & Sons Editions

[B23] Gwatkin DR Rutstein S Johnson K Suliman E Wagstaff A Amouzou A Socio-economic differences in health, nutrition, and population within developing countries: an overview Niger J Clin Pract 2007 10 272 282 18293634

[B24] Vyas S Kumaranayake L Constructing socio-economic status indices: how to use principal components analysis 2006 Oxford University Press in association with The London School of Hygiene and Tropical Medicine 459 468 10.1093/heapol/czl029 17030551

[B25] Nyarango RM Aloo PA Kabiru EW Nyanchongi BO The risk of pathogenic intestinal parasite infections in Kisii Municipality, Kenya BMC Public Health 2008 8 237 10.1186/1471-2458-8-237 18620608 PMC2478685

[B26] Shahnazi M Jafari-Sabet M Prevalence of parasitic contamination of raw vegetables in village of Qazvin province, Iran Foodborne Pathog Dis 2010 7 1025 1030 10.1089/fpd.2009.0477 20491596

[B27] Amoah P Drechsel P Abaidoo RC Klutse A Effectiveness of common and improved sanitary washing methods in selected cities of West Africa for the reduction of coliform bacteria and helminth eggs on vegetables Tropical Medicine & International Health 2007 12 Suppl. 2 40 50 18005314 10.1111/j.1365-3156.2007.01940.x

[B28] Feachem RG Bradley DJ Garelick H Mara DD Sanitation and Disease: Health Aspects of Excreta and Wastewater Management 1983 New York: John Wiley & Sons Editions

[B29] Wittnich C *Entamoeba histolytica *Infection in a German shepherd dog Can Vet J 1976 17 259 263 184910 PMC1697359

[B30] Shimada S Muraki Y Awakura T Umemura T Sanekata T Kuroki T Ishihara M Necrotic colitis associated with *Entamoeba histolytica *infection in a cat J Comp Pathol 1992 106 195 199 10.1016/0021-9975(92)90048-Y 1597535

[B31] Jensen PK Phuc PD Dalsgaard A Konradsen F Successful sanitation promotion must recognize the use of latrine wastes in agriculture - the example of Vietnam Bull World Health Org 2005 83 273 274 PMC2626463 16302046

[B32] Khuong NC Phuc PD Bich TH Hung N-V Health Risks from Excreta and Wastewater to Vietnamese Farmers Sandec News 2010 11 7

[B33] Raschid-Sally L Doan-Tuan D Abayawardana S National assessment on wastewater use in agriculture and an emerging typology: Vietnam case study. Wastewater use in irrigated agriculture: confronting the livelihood and environmental realities Cabi Publishing, Wallingford 2004

[B34] Fuchs G Ruiz-Palacio G Pickering LG Ravdin JI Amoebiasis in the pediatric population Amoebiasis: human infection by Entamoeba histolytica 1988 New York: John Wiley and Sons 594 613

[B35] Ravdin JI Amebiasis Clin Infect Dis 1995 20 1453 1464 10.1093/clinids/20.6.1453 7548493

[B36] Tellez A Morales W Rivera T Meyer E Leiva B Linder E Prevalence of intestinal parasites in the human population of Leon, Nicaragua Acta Trop 1997 66 119 125 10.1016/S0001-706X(97)00037-5 9210962

[B37] Shakya B Rai SK Singh A Shrestha A Intestinal parasitosis among the elderly people in Kathmandu Valley Nepal Med Coll J 2006 8 243 247 17357641

[B38] Siddiqui MI Bilqees FM Iliyas M Perveen S Prevalence of parasitic infections in a rural area of Karachi, Pakistan J Pak Med Assoc 2002 52 315 320 12481664

[B39] Blessmann J Van LP Nu PA Thi HD Muller-Myhsok B Buss H Tannich E Epidemiology of amebiasis in a region of high incidence of amebic liver abscess in central Vietnam Am J Trop Med Hyg 2002 66 578 583 12201594 10.4269/ajtmh.2002.66.578

[B40] Rukmanee P Wuthisen P Thanyavanich N Puangva-art S Rukmanee N Factors associated with intestinal parasites among households in Ratchaburi province, Thai-Myanmar border area J Trop Med Parasitol 2008 31 85 94

[B41] Haque R Ali IK Akther S Petri WA Comparison of PCR, isoenzyme analysis, and antigen detection for diagnosis of Entamoeba histolytica infection J Clin Microbiol 1998 36 449 452 9466756 10.1128/jcm.36.2.449-452.1998 PMC104557

[B42] Markell EK John DT Krotoshi WA Lumen dwelling protozoa 1999 8 Mexico: Saunders Company 24 89

